# Unveiling functional heterogeneity in breast cancer multicellular tumor spheroids through single-cell RNA-seq

**DOI:** 10.1038/s41598-020-69026-7

**Published:** 2020-07-29

**Authors:** Erick Andrés Muciño-Olmos, Aarón Vázquez-Jiménez, Ugo Avila-Ponce de León, Meztli Matadamas-Guzman, Vilma Maldonado, Tayde López-Santaella, Abrahan Hernández-Hernández, Osbaldo Resendis-Antonio

**Affiliations:** 1 PhD Program in Biomedical Sciences, UNAM, Mexico City, Mexico; 2 Human Systems Biology Laboratory, Instituto Nacional de Medicina Genómica, Periférico Sur 4809, Arenal Tepepan, 14610 Mexico City, Mexico; 3 PhD Program in Biological Sciences, UNAM, Mexico City, Mexico; 4 Biología de Células Individuales (BIOCELIN), Laboratorio de Investigación en Patología Experimental, Hospital Infantil de México Federico Gómez, Mexico City, Mexico; 5 Epigenetic Laboratory, Instituto Nacional de Medicina, Genómica, Periférico Sur 4809, Arenal Tepepan, 14610 Mexico City, Mexico; 6 Coordinación de La Investigación Científica -Red de Apoyo a La Investigación, UNAM, Mexico City, Mexico

**Keywords:** Cancer, Computational biology and bioinformatics, Systems biology

## Abstract

Heterogeneity is an intrinsic characteristic of cancer. Even in isogenic tumors, cell populations exhibit differential cellular programs that overall supply malignancy and decrease treatment efficiency. In this study, we investigated the functional relationship among cell subtypes and how this interdependency can promote tumor development in a cancer cell line. To do so, we performed single-cell RNA-seq of MCF7 Multicellular Tumor Spheroids as a tumor model. Analysis of single-cell transcriptomes at two-time points of the spheroid growth, allowed us to dissect their functional relationship. As a result, three major robust cellular clusters, with a non-redundant complementary composition, were found. Meanwhile, one cluster promotes proliferation, others mainly activate mechanisms to invade other tissues and serve as a reservoir population conserved over time. Our results provide evidence to see cancer as a systemic unit that has cell populations with task stratification with the ultimate goal of preserving the hallmarks in tumors.

## Introduction

Cancer studies have established that tumors are complex and heterogeneous systems. These properties are grounded on genetic variations and diverse microenvironmental conditions that induce sizable differences in gene expression profiles, surface biomarkers, metabolism, growth rates, morphology, metastatic potential and response to therapy at a single cell level^1,2^. From a clinical point of view, intratumoral (inside tumors) and intertumoral (between tumors) heterogeneity are critical factors that influence diagnosis outcomes and treatments in patients^1,3^. Given their relevance, the understanding of tumor heterogeneity has emerged as a fundamental aim to improve treatment efficiency^4^.

To portray cancer intratumoral heterogeneity in human tissues, tumor microenvironment and their cellular population have been depicted in genome atlases for different cancer types^5–7^. Notwithstanding the relevance of these atlases, big challenges into experimental designs come across to survey heterogeneity in human biopsies. For instance, proper cell dissociation methods must be implemented to reduce the risk of altering the transcriptional landscape^8^. Additionally, it is hard to trace tumor dynamics due to invasive procedures and the inherent risk to patients^9^. Interestingly, to overcome previous limitations, xenograft and organoid models have been used to emulate the temporal and three-dimensional organization of complex cell populations^10^.

On the other hand, intratumoral heterogeneity is an intricate property that influences even isogenic models providing complementary prosurvival functional roles, called functional heterogeneity^11,12^. Functional heterogeneity is hard to be evaluated in the previously described models because of tumor complex interactions^9^. To get the functional heterogeneity basics and design optimal treatments to overcome cancer, in vitro studies are the gold standard to build a comprehensive assessment of cancer development. Additionally, they are one of the best options to perform longitudinal studies. This approach has been used to study glioblastoma clonal drug resistance by the cooperation of two sensitive clones^12^. In vitro models establish a valuable platform for the experimental assessment of testable hypotheses emerging from quantitative approaches, particularly those related to selected mechanisms associated with therapeutic applications^13^.

Conversely, monolayer in vitro models lack the three-dimensional organization that is fundamental in cancer^9^. Otherwise, multicellular tumor spheroids (MCTS) are scaffold-free self-assembled aggregates of isogenic cancer cells displaying an intermediate complexity between monolayer cell cultures and in vivo solid tumors^14^. MCTS develop a heterogeneous microenvironment that leads to the generation of proliferative, quiescent, and necrotic/apoptotic subpopulations^15,16^. This in vitro cancer model is valuable as they mimic the first avascular stages of tumor formation, and exhibit aggressiveness features such as multicellular resistance, migration, and invasion^17^. Additionally, it provides an experimental framework to carry out tumor growth, hypoxia, migration-invasion, drug screening, and radiosensitivity studies^15,18^. Furthermore, the similarities between MCTS and tumors, plus their experimental advantages, such as reproducibility and evaluations at several time points, makes them an appealing model to unravel their heterogeneous composition of clonal subpopulations at different stages of growth and figure out their functional interrelationship.

In this paper, we present the analysis of the functional heterogeneity that promotes tumor survival for an in vitro model. To this end, we used the MCF7 cell line, a well-established in vitro model of invasive luminal A subtype of breast cancer with HR+/HER2- phenotype, which has been useful to dissect breast cancer mechanisms^19–21^. On top of that, luminal A subtype represents the most frequent cancer among women worldwide^22–25^. To characterize functional heterogeneity in tumor development, we carried out a two-time points study during the growth of MCF7 MCTS. Our experimental design allowed us to obtain the gene expression profile of 364 single cells collected at 6 and 19 days of growth. To describe undergoing functional pathways and cellular processes for each cell subpopulation, we performed an integrative data-driven approach combining clustering algorithms, single-cell differential gene expression analysis, and gene set enrichment analysis. Our data revealed that despite the temporal progression of MCTS, they were permanently integrated by three major cellular subpopulations with complementary functional properties: proliferative, invasive/evasive, and a reservoir. Therefore, we postulate that self-organized subpopulations emerge as functional stratification mechanisms to ensure tumor progression. This single-cell study on MCF7 provides the foundations for viewing cancer as a collaborative ecosystem that drives different and complementary hallmarks enhancing and promoting tumor survival during the temporal evolution of the tumor.

## Results

### Polarized processes through biomarkers expression

**Figure 1:**
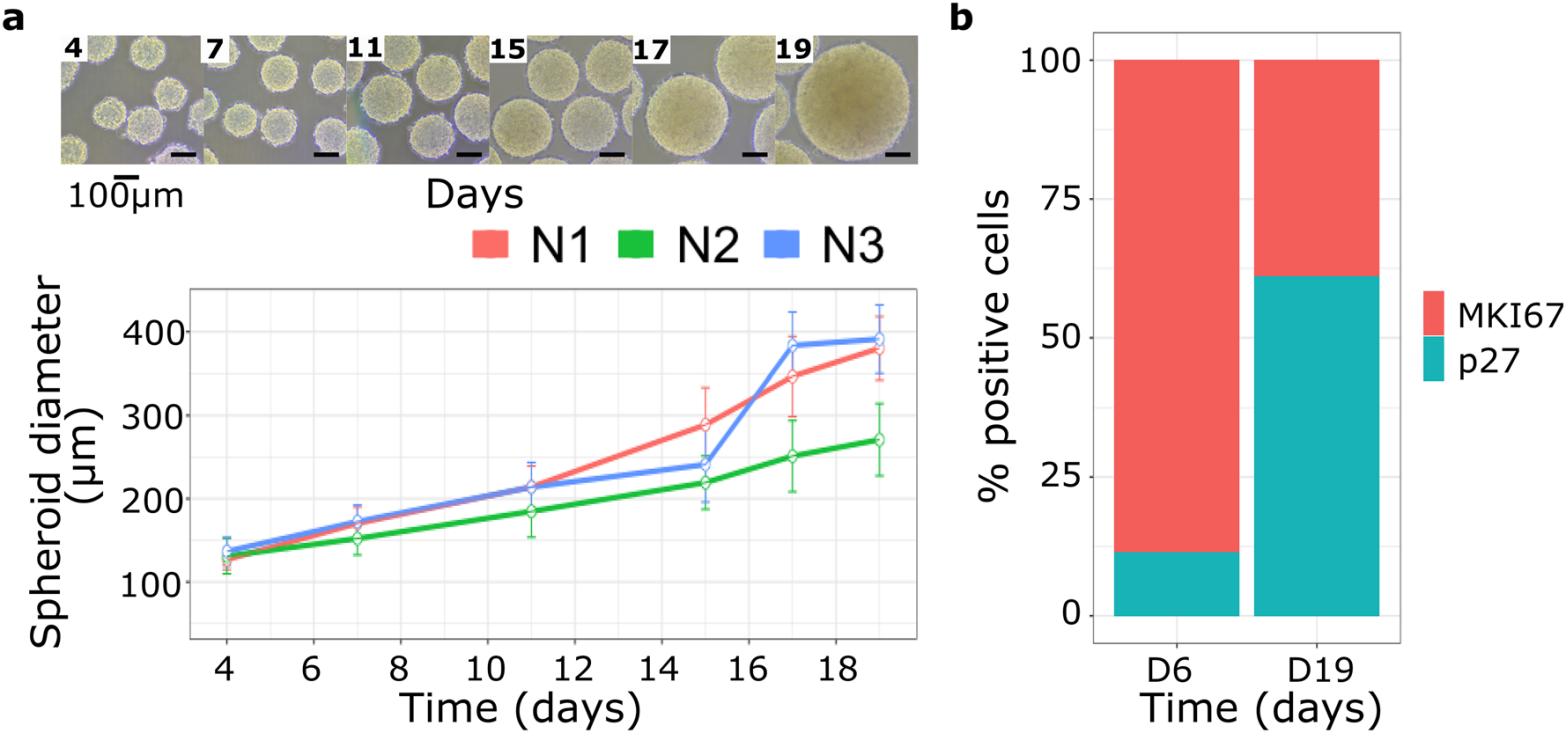
(**a**) Multicellular Cancer Tumor Spheroids of breast cancer cell line MCF7 diameter along time. We performed three independent biological replicates of the MCF7-MCTS culture kinetic denoted as N1, N2 and N3. Points and bars represent the average Feret diameter with their respective standard deviation for each replicate and time-point. Values can be found in Supplementary Table S1. (**b**) Immunophenotyping with flow cytometry analysis measuring MKI67 and p27 biomarkers at days 6 and 19, expression of both markers for the two-time points showed statistical difference with a *p* value < 0.05 using a non-paired t-student test with equality of variances.

MCF7 multicellular tumor spheroids (MCF7 MCTS) allowed us to study in vitro the relationship between functional heterogeneity and tumor progression. MCF7 MCTS were cultured for 19 days, and their diameter distribution was measured by image analysis of photos taken to the culture flask at three independent culture kinetics in different days of progression. Average Feret diameter and their respective standard deviation are shown for every time-point and replicate (Fig. 1a). For each measurement, the number of quantified spheroids varied from 16 to 116, with an average diameter ranging between 126 and 391 μm (Supplementary Table S1). In all cases, measured diameters did not exceed 450 μm, the limit-size that spheroids can reach with a low rate of necrotic cores produced due to oxygen and nutrient gradients; nevertheless, samples from the 19th day still had necrotic cores but qualitatively in less proportion^26^.

As can be seen in Fig. 1a, MCTS diameter increased linearly with time. As tumors increase in size, cellular composition changes as a natural evolution of the disease. In order to survey tumor heterogeneity over time, we evaluated the expression of MKI67 and p27. MKI67 is a common proliferation biomarker expressed only during active phases of the cell cycle^27^, whereas p27 is a cell cycle arrest biomarker^28^. MCTS were properly aggregated on day 6 and reached their growth capabilities on day 20. After day 20, almost all spheroids developed necrotic cores, as already established^26^. Using immunophenotyping with flow cytometry, we found major differences of MKI67 and p27 biomarkers between the 6-day (D6) and the 19-day (D19) spheroids (Supplementary Figure S1). We noticed that almost all cells conforming D6 spheroids overexpressed MKI67 compared with D19 spheroid cells. On the contrary, D19 cells overexpressed p27 (Fig. 1b). Our data showed that our MCTS model comprised two subpopulations with differences in their functionality over time. Therefore, based on the expression of p27 and MKI67, we found a proliferative enriched tumor and a quiescent enriched tumor in D6 and D19 spheroids, respectively. To get more insights about this heterogeneity and the transcriptional differences in subpopulations, we performed single-cell RNA-seq of D6 and D19 MCF7 MCTS.

### Insights of functional heterogeneity

**Figure 2:**
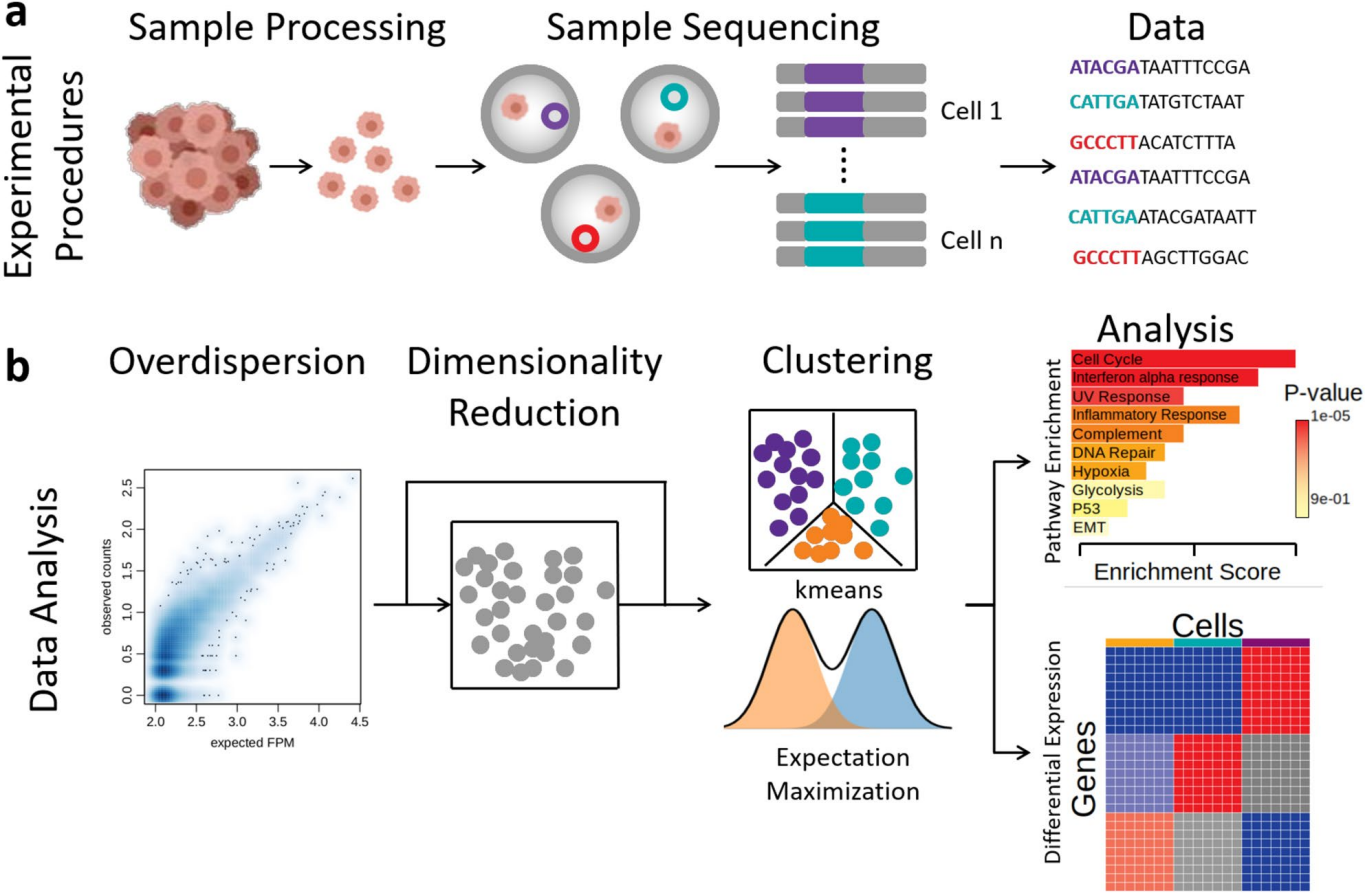
Methodological layout. (**a**) Experimental procedure to obtain single-cell transcriptome data from MCTS. (**b**) Data analysis pipeline followed to obtain the functional heterogeneity description.

To elucidate the connection between subpopulation composition and task diversification in tumors, we performed single-cell RNA-seq from MCF7 MCTS at 6 and 19 days of development. First, MCTS were disaggregated, filtered to exclude dead cells, encapsulated in drops, and the RNA of isolated single cells was labeled. Then, next-generation sequencing technology was applied to obtain single-cell gene expression profiles (13,589 genes) of 364 cells in both time points. Secondly, to obtain ongoing cellular processes, the overdispersion of gene expression for all cells was computed, and cells were clustered without a predefined number of clusters. Finally, we applied gene set enrichment and differentially expression analyses to describe and characterize functional heterogeneity. The methodological layout followed is depicted in Fig. 2.

#### Clustering single-cell data to identify subpopulations in MCF7 MCTS

**Figure 3:**
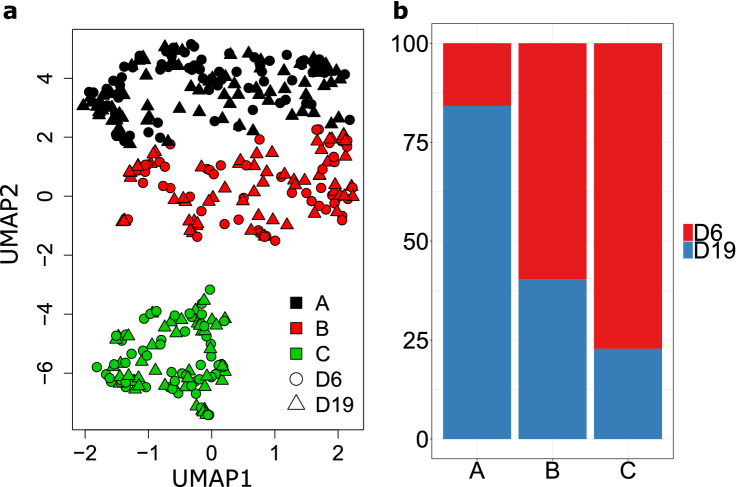
(**a**) Data clustering using K-means algorithm. Three clusters (black, red, green points) were identified and represented on a 2D UMAP plot. Circles and triangles represent the sample time of days 6 and 19, respectively. (**b**) Normalized proportion of cells from D6 and D19 time points in each cluster.

Aiming to identify subpopulations in MCTS, the distance matrix of overdispersion was projected into the bidimensional UMAP space (Uniform Manifold Approximation and Projection)^29^ (Fig. 3a). Dimensionality reduction projection suggested that there is no separation between time samples. Therefore, we combined all the data and grouped using the K-means clustering algorithm. By doing so, cells with similar characteristics were grouped despite differences in time samples. Three clusters, each one with different proportions of cells from D6 and D19 samples (Fig. 3b). Cluster A had around 85% of D19 cells, Cluster C around 80% of D6 cells, and cluster B had a homogeneous mix of D6 and D19 cells. Consequently, the three subpopulations coexisted throughout MCTS development. Although the clustering methods established similarities and differences in data, there was no information about the functional roles of cells grouped in each cluster.

#### Characterization of clusters and mapping their biological functionality

We evaluated the functional composition across clusters by pathway enrichment analysis. Using the GSEA tool^30^, we evaluated the correlation between our data and established gene sets to find overrepresented biological pathways. For Cluster A the number of positive enriched pathways was 344 with an FDR < 0.05 and a *p* value < 0.01. Supplementary Figure S2 shows the barplot for the 15 enriched gene sets of the MSigDB hallmark collection; interestingly, results suggest an activation of the immune system response. See Supplementary Table S2 for a full list of the enriched pathways.

**Figure 4:**
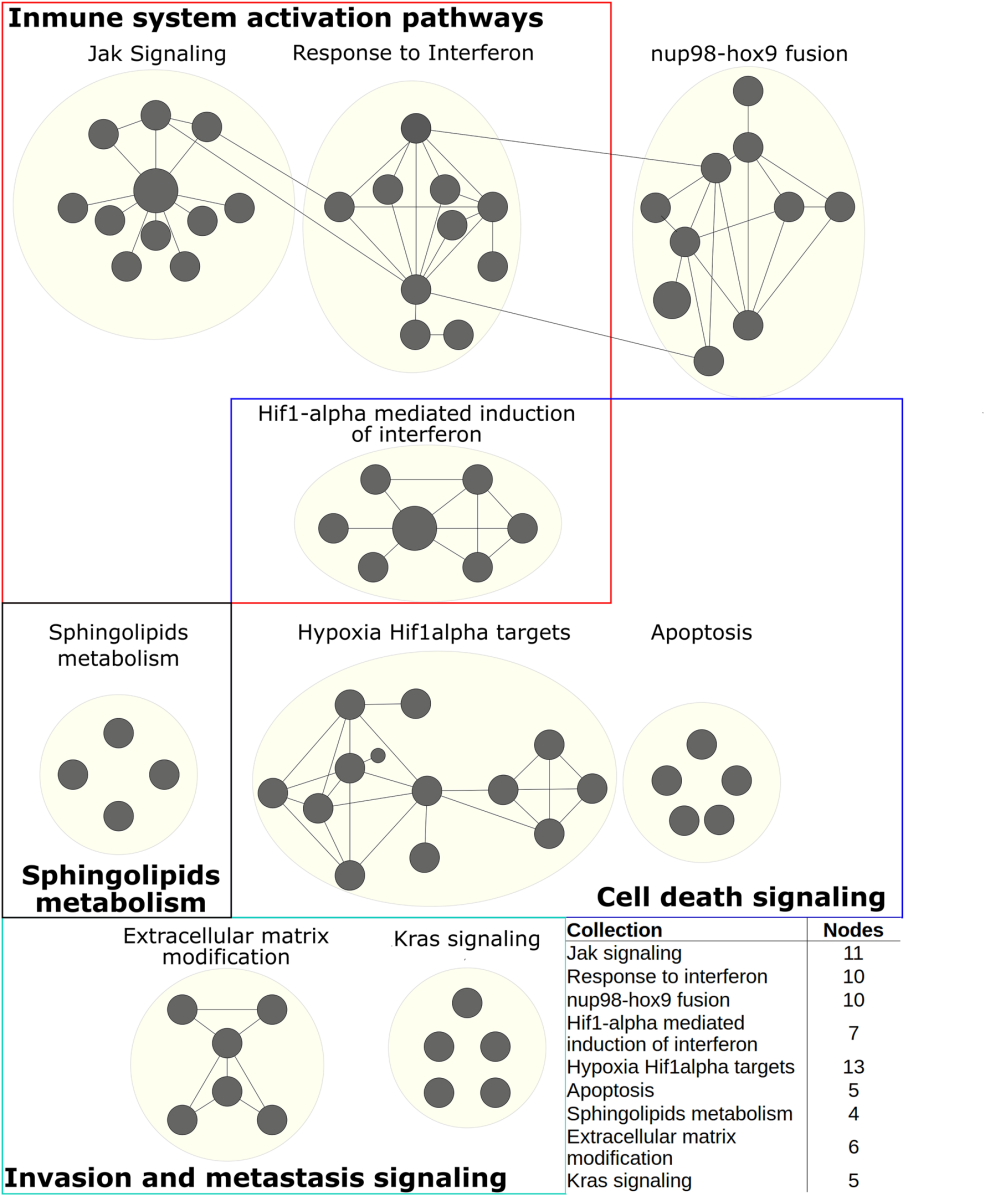
Positive enriched map for Cluster A. Each black circle (node) represents a pathway, circle size represents the number of genes represented between the gene sets and data. Links between pathways were established when 2 pathways had a Jaccard index > 0.4, given the expressed genes. Colour squares denote a category that comprises several collections (yellow circles) with shared biological function. The embedded table shows the number of pathways enriched in each collection. All the enriched pathways had an FDR < 0.05 and a *p* value < 0.01.

In order to visualize and capture the global scope of the activated biological functions positively associated with Cluster A, we built a network based on the enriched pathways and the number of genes shared between them. We focused our analysis on pathways that shared a fraction of genes among them. Therefore, 71 of the total genesets were associated into different collections describing general processes. Enriched pathways for every collection are shown in Supplementary Table S3. Figure 4 shows the enriched map for the overall positive enriched pathways to Cluster A compared with the other two clusters. Every node represents an enriched pathway, and its size indicates the number of overexpressed genes in the specific geneset. To represent interconnectivity and genes overlapping between pathways, we computed the Jaccard index between the set of genes integrating each pair of pathways. When the Jaccard index was above 0.4, we connected the nodes indicating that both pathways shared at least 40% of their genes. Finally, yellow circles represent a common process by the association of similar enriched pathways in higher-level processes given a specific biological function.

Overall enriched pathways for Cluster A were summarized in four categories: (1) Activation of immune processes. (2) Sphingolipids metabolism. (3) Invasion and metastasis; and (4) Cell death signaling pathways. The Activation of immune processes category had 28 overexpressed gene sets that mainly enclosed genes activated by the interferon pathway, HIF1-alpha, and the overactivation of the Jak signaling pathway. The Sphingolipids Metabolism category had four pathways, and it had the gene SPHK1 as a leading-edge gene. In concordance with this result, there is evidence that human breast cancer presents a dysregulation in sphingolipids metabolism^31^. Particularly, SPHK1 protein catalyzes the phosphorylation of sphingosine process associated with the mediation of pro-survival signaling and metastasis^32^. The Invasion and Metastasis category had 11 overexpressed gene sets that were associated with the extracellular matrix modification pathways and the activation of the KRAS signaling pathway. Finally, the Cell Death category comprised pathways of hypoxia and apoptosis. The Nup98-hox9 process has not been categorized. Besides, dysregulation of HOX genes can occur due to their fusion to nucleoporin (NUP98) and has been associated with malignant transformation^33^. Given our findings, we suggest Cluster A might has an invasive and pro-survival role.

**Figure 5:**
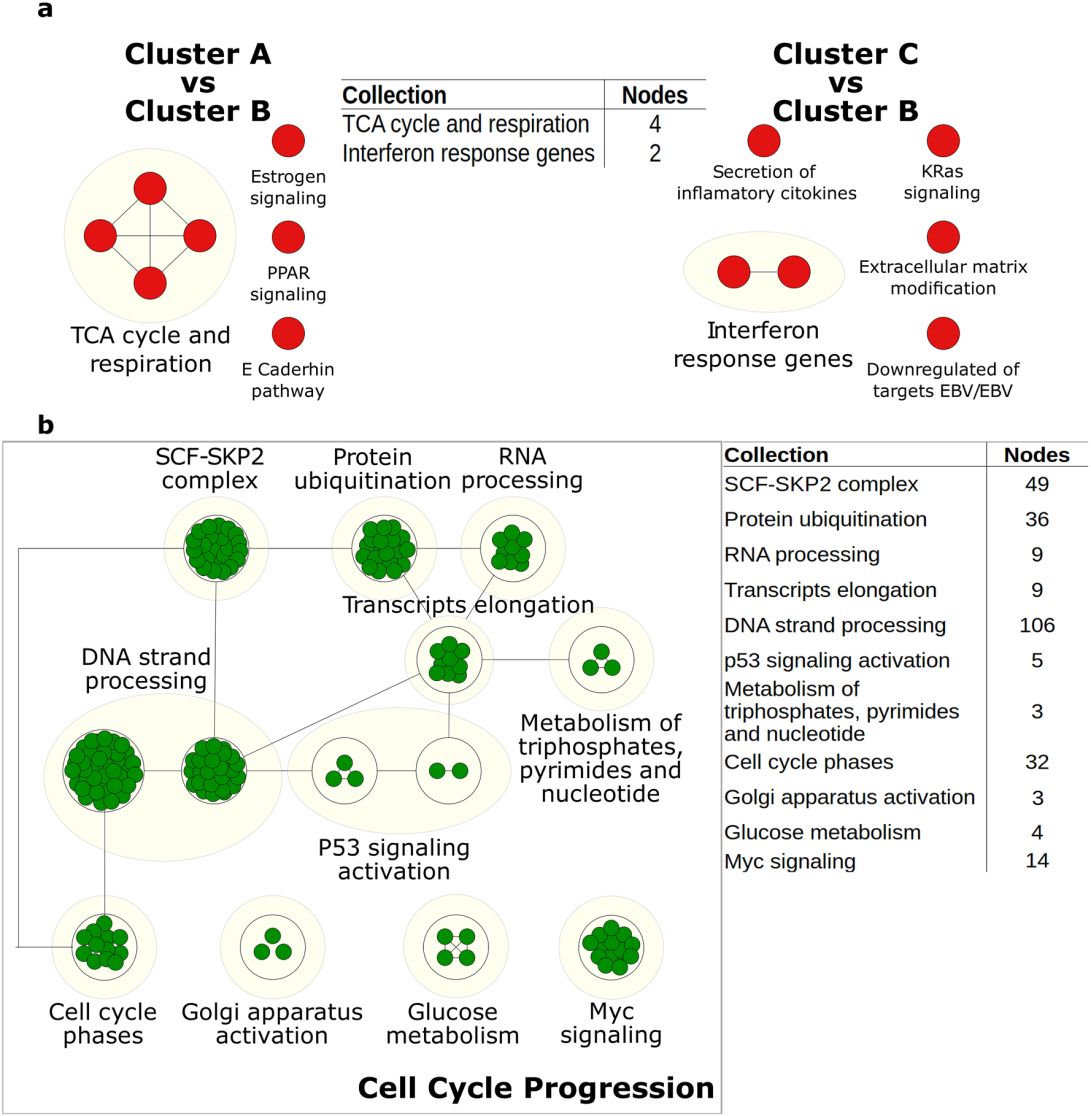
Positive enriched maps, each green and red circles are nodes that represent a pathway. Yellow circles related a collection with a biological function; tables show the number of pathways enriched in every collection. Links between pathways were established when 2 pathways or collections had a Jaccard index > 0.4 by the expressed genes. All the enriched pathways have an FDR < 0.05 and a *p* value < 0.01. (**a**) Enriched pathways for cluster B given the paired-comparison with other clusters. (**b**) Enriched pathways for cluster C, black square associate all collections with a high-end biological process.

Cluster B enrichment only showed an overall pathway related to interferon responsive genes. Therefore, to explore and associate a particular phenotype and identify an ongoing process, we performed pathway enrichment by pairs (Fig. 5a and Supplementary Figure S3). Enrichment analysis for Cluster B versus Cluster A showed seven positive correlated pathways with an FDR < 0.05 and *p* value < 0.01. Under this comparison, there is an overactivation of the TCA cycle and electron transport chain genes. Also, we observed an enrichment of genes related to transcriptional activation under estrogens stimuli and peroxisome proliferator-activated receptors (PPAR). Finally, cell adhesion processes might be taken part in this cluster as E-cadherin is typically expressed in epithelial cells^34^. On the other hand, Cluster B vs Cluster C comparison had 6 positive enriched pathways. Cluster B displayed similar results to Cluster A, such as the activation of genes related to interferon and cytokines secretion. In addition, pathways of extracellular matrix modification and KRAS were overexpressed. Despite that enriched gene sets are limited due to Cluster B similarities in their gene expression profiles with the other clusters, the variability in data cannot be explained just by two clusters hence Cluster B is important to be considered as a unique subgroup. According to the polarized results of Cluster B, we hypothesize the existence of a transition subpopulation or reservoir with a backup function. All the positive enriched pathways are shown in Supplementary Table S4, Supplementary Table S5 and Supplementary Figure S3.

**Figure 6:**
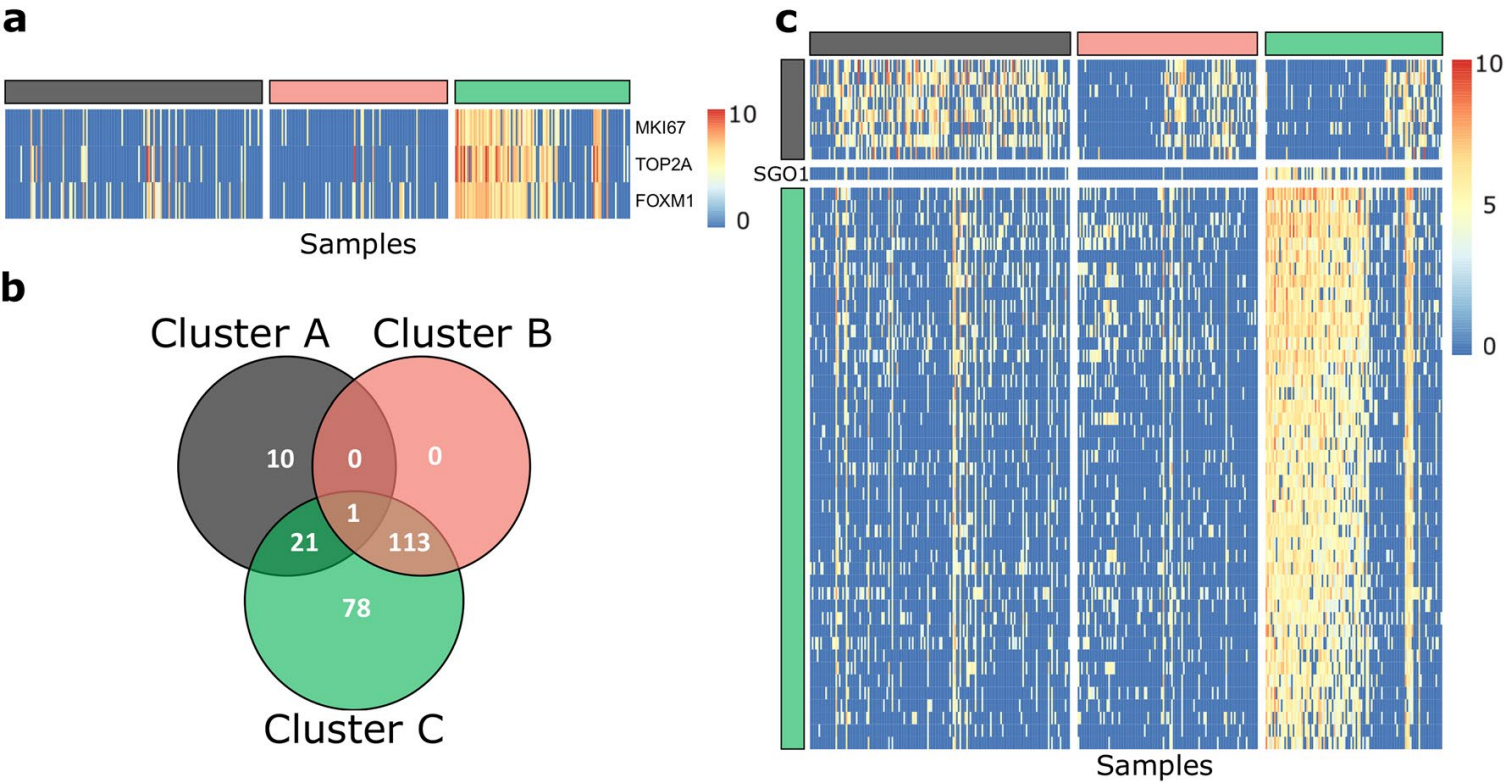
Differentially expressed genes. (**a**) Heatmap of genes TOP2A, MKI67, and FOXM1. Log fold change > 1.5 and *p* < 0.05. Colors yellow and red represent the low and high expression, respectively. (**b**) Venn diagram for the differentially expressed genes for every cluster. c. Heatmap of the marker genes for each cluster with a |log2(FC)|> 2 and a *p* < 0.01 (empirical *p* value estimation). Rows are the markers genes for every cluster and columns are the cluster samples.

Regarding Cluster C, we found 445 positive enriched pathways with an FDR < 0.05 and a *p* value < 0.01, 274 of them belong to 11 collections that point to one big category: cell cycle progression. Supplementary Figure S4 shows the barplot for the 22 enriched gene sets of the hallmarks and KEGG MSigDB collections, the full list of the enriched pathways and the enriched pathways for every collection are presented in Tables S6 and S7. Interestingly, Cluster C enriched map and their pathways collections (Fig. 5b) showed upregulation of every phase of the cell cycle, compared to clusters A and B. Therefore, processes like DNA replication, transcription, and translation with their particular maturation were happening in Cluster C cells. In agreement, genes regulated by p53 and Myc have differential roles linked to cell proliferation. This cluster seems to be metabolizing macromolecules like glucose and phosphate bases. It supplies evidence of a direct relationship between cell cycle activation/progression and metabolism of amino acids, purine, and pyrimidine. Consequently, there are insights that Cluster C cells were proliferating. We confirmed the expression of MKI67, FOXM1, and TOP2A as proliferation markers^35^ in our gene expression data for Cluster C (Fig. 6a). Altogether, we identified that clusters A and C were associated with specific functional pathways, while Cluster B remained as an undefined group.

#### Differential gene expression analysis

To identify differentially expressed genes on the three clusters previously described, expression of each gene in every cluster was compared pairwise and filtered using a |log2(FC)|> 1.5 and a *p* value < 0.01. Figure 6b shows the Venn diagram using the results of every comparison (Supplementary Table S8). As a result, we found a total of 223 up and downregulated genes corresponding to 3.71% of the entire set of evaluated genes. Among them, the expression profile of 89 genes was specific for clusters and not conditional for a specific pairwise comparison. These genes may be candidates to be markers genes of each cluster. In particular, we found that SGO1, a gene related to centromeric cohesion in mitosis^36^, is differentially expressed in all clusters (center of the Venn diagram). Figure 6c shows the landscape of the 55 overexpressed genes with the most significant log-ratios among clusters (|log2(FC)|> 2, Supplementary Table S9).

### Key genes in functional heterogeneity

So far, enriched pathways gave us insight into particular processes, and the differentially expressed analysis allowed us to know which genes had the highest expression among clusters. To be able to set the differentially expressed genes that govern the enriched pathways, we linked the above results. We matched the differentially expressed genes and the leading-edge genes with the most recurrence in collections described in the enriched maps for each cluster (Figs. 4 and 5).

**Figure 7:**
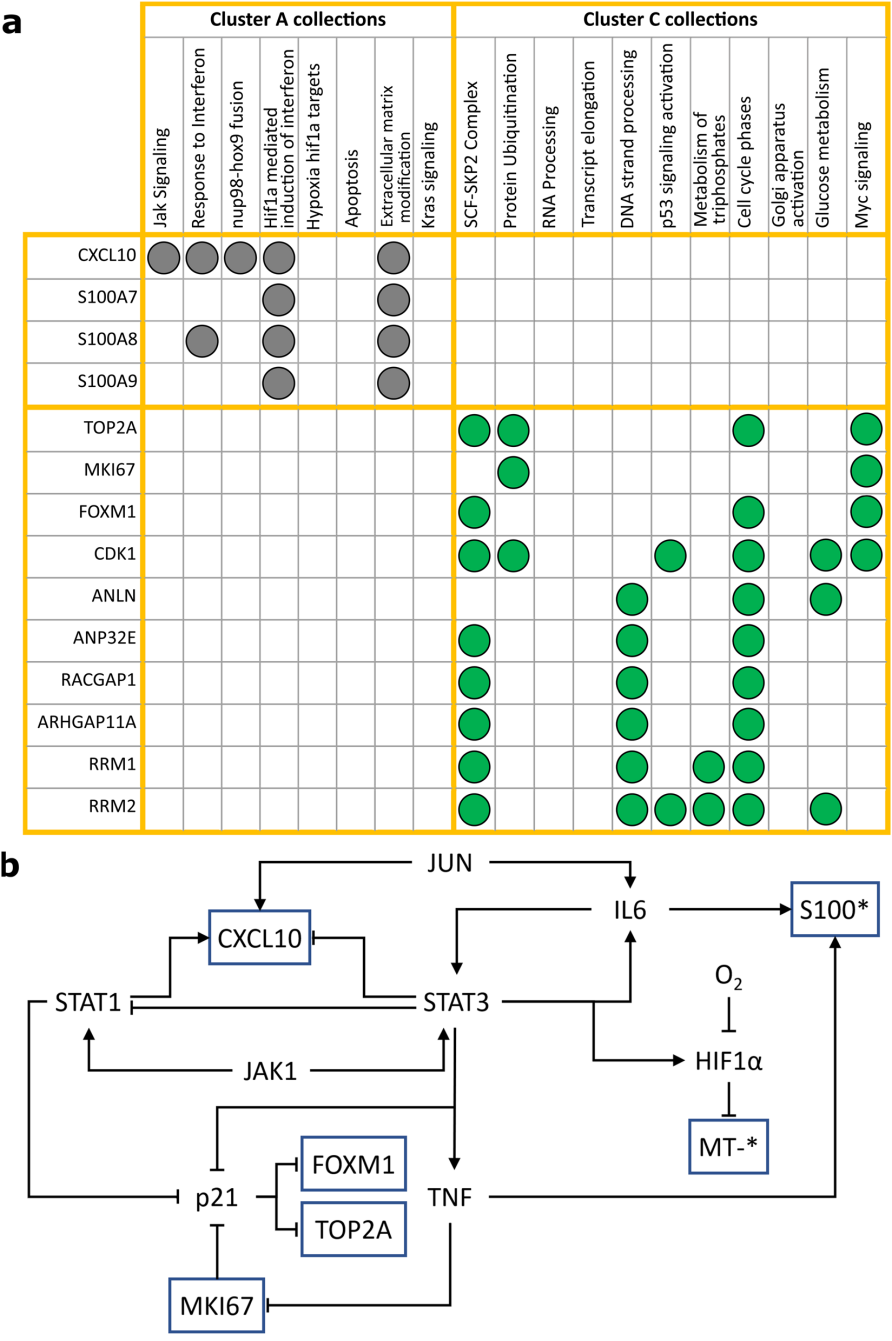
(**a**) Association graph between differentially expressed genes (rows) and the collections obtained for the enriched maps. Circles indicate strongly enriched genes in a collection. Gray and green circles represent collections for clusters A and C, respectively. (**b**) Proposed interaction mechanism based on literature association with IPA. Arrows indicate activation and bars inhibition. The genes in blue squares are the ones overexpressed in the clusters. S100* encloses S100A7, S100A8 and S100A9. MT-* encloses genes related to respiratory chain (MT-CO1, MT-CO2, MT-CO3, MT-RNR1, MT-ND4L, MT-ND5, and MT-ND2).

Figure 7a shows the differentially expressed genes for cluster A and C that correlates with the enriched collections. The most overrepresented gene for Cluster A was CXCL10, which shapes the enrichment core for several associated collections: extracellular matrix modification, Jak signaling, response to interferon, nup98-hox9 fusion, and HIF1-alpha mediated induction of interferon (Figs. 4 and 7a). In summary, these functions described immune and migration-related processes. For instance, CXCL10 and its receptor CXCR3 play an essential role in metastasis in various cancer cells, including colorectal carcinoma cells, colon cancer, prostate cancer, melanoma, and glioma^37^. Moreover, CXCL10 is overexpressed in breast cancer, and their expression is positively correlated with advanced tumor stages^38^. Additionally, S100 genes (S100A7, S100A8, and S100A9) were pointed out as important according to our results, particularly S1008. They are associated with several Cluster A collections: response to interferon (S100A8), Hif1a mediated induction of interferon (S100A7, S100A8, and S100A9) and extracellular modifications (S100A7, S100A8, and S100A9). These genes may represent a link between inflammatory and metastatic processes. Even though these proteins are usually expressed in myeloid cells, such as neutrophils and macrophages, there is experimental evidence showing their overexpression in breast cancer^39^. We could not find a key gene in Cluster B because it was poorly pathway enriched and only had one differentially expressed gene.

Cluster C had well-known proliferation markers (MKI67, TOP2A, FOXM1, CDK1) as part of the enrichment core for this cluster (Figs. 6 and 7a). Moreover, ANLN, ANP32E, RACGAP1, and ARHGAP11A were also differentially expressed and are implicated in proliferation. Nevertheless, ANLN overexpression increases the migratory capacity of cells in breast cancer^40^. ANP32E-miR-141 axis participates in the regulation of cell proliferation, migration, and invasion in breast cancer cell lines^40,41^. RACGAP1 inhibits cell migration in Basal-like Breast Cancer cell lines, while ARHGAP11A promotes it^42^. Furthermore, other relevant genes are RRM1 and RRM2 due to their correlation to the metabolism of triphosphates. Both genes are the catalytic subunits of ribonucleotide reductase (RNR) that set the limiting step in dNTP synthesis, a process needed for proliferation. To expand this result, Cluster C cells overexpressed mitochondrial genes that are part of the respiratory complexes (MT-ND3, MT-ATP6, MT-CO2, MT-ND4, MT-CYB, MT-CO3, MT-ND2, MT-CO1, MT-ND5, and MT-RNR1) related to mitochondrial oxidative phosphorylation (OXPHOS). Therefore, these observations suggest a metabolic rewiring among this subpopulation, needed to meet energy demands in the cancer cell^43,44^.

In conclusion, we found genes in subpopulations that might rule some cancer hallmarks in MCTS, ranging from metabolic rewiring to global functions like proliferation and migration. Our results highlight the question if there is a motif capable of regulating all cluster processes.

### Depicting a regulation motif

To investigate if the previously described genes are interconnected between them and propose a possible genetic circuit that modulates MCF7 MCTS subpopulations, we identified upstream regulators interconnected with several identified genes, using the upstream regulators tool in Ingenuity Pathway Analysis software (IPA). So, we only considered mitochondrial genes, S100 family genes, CXCL10, FOXM1, TOP2A, and MKI67. As a result, we found eight upstream interacting genes (HIF1A, STAT1, STAT3, IL6, p21, JUN, TNF, and JAK1) and oxygen that might be the central regulation core. The motif with their regulatory associations is depicted in Fig. 7b. Arrows and bar-end connectors indicate activation and inhibition, respectively. Measured genes are shown in blue contour. Notably, most of these genes are cancer master regulators, and here we supply evidence that all subpopulations together shape a functional heterogeneity core.

### Robustness of the functional heterogeneity

Previous results indicated functional heterogeneity among cell subgroups. To discard an induced bias due to dimensionality reduction and clustering methods, we repeated the analysis using the entire multivariable space and expectation–maximization (EM) clustering algorithm. EM is an unsupervised nonparametric method of classification that does not define a priori the number of clusters in the samples^45^. To ensure robust physiological interpretations, we applied four combinatorial analyses by considering two clustering algorithms (K-means and EM) into the full variance space (Var) and the UMAP projection. Each gene set enrichment analysis was performed with the same gene sets collection. Table 1 shows the number of enriched pathways with an FDR < 0.05 and a *p* value < 0.01 for all analyses, no additional restrictions were set for the enrichment score.

**Table 1:**
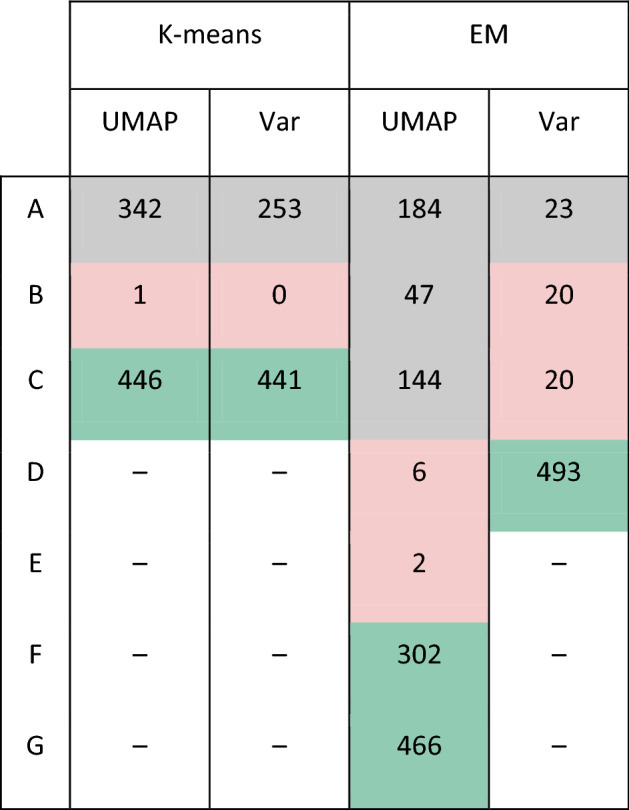
Number of enriched pathways by GSEA tool. Two clustering methods were used (K-means and EM) in two spaces, one dimensionally reduced (UMAP) and another without any reduction process (Var). The numbers represent the number of enriched pathways (FDR < 0.05 and *p* value < 0.01). Ndash symbol (–) represents the non-existence of that particular cluster. Gray, red, and green associate clusters with similarities based on the enriched pathways given a Jaccard Index equal to 0.7.

As Table 1 depicts, there are differences in the number of enriched pathways obtained for each combination of projected space and clustering methods. Meanwhile, UMAP and K-means suggested the presence of three main clusters, the number of clusters obtained from the EM algorithm using UMAP space was seven. Despite the EM algorithm being able to identify more clusters than K-means, we noted that the enriched pathways obtained suggests a functional correspondence between methodologies. To compare our previous findings with this new classification, we calculated the degree of association among clusters through the Jaccard index of the enriched pathways. Jaccard index was empirically fixed at 0.7 because it was the highest value where associations were found. Despite the different number of clusters obtained for each approach, we found consistency between them. For instance, clusters A, B, and C in EM-UMAP analysis correspond to A in the K-means analyses. D and E are related to B, and Clusters F and G are correlated to C. As Cluster B does not have a particular enriched pathway, we associated clusters with an undefined function. Table 1 shows the clusters obtained for each method, and colors indicate similar functional subpopulations; gray, red, and green cells correspond to the invasive, reservoir, and proliferation subpopulations, respectively. So, despite the differences in the number of clusters, the results described do not change, and it is possible to extract similar conclusions. These findings give us the confidence that results do not depend on a particular method.

On the other hand, to ensure the robustness of these findings we validated our results on a parallel study applying RNAseq with the same experimental setup. Despite the differences in their scope, we concluded that in general terms, both technologies are in agreement to identify crucial processes being altered during the spheroid progression, such as immune systems activation, cell cycle progression, and proliferative pathways (data not shown).

In conclusion, MCF7 MCTS single-cell analysis showed that isogenic cancer cells have different functions to ensure tumor progression. Determining that functions among cancer cells within a tumor is important to know why some cancer cells are resistant to treatments, causing cancer relapses in patients.

## Discussion

With the purpose to unveil functional heterogeneity during tumor progression, we examined the transcriptome of single cells from MCF7 MCTS at two-time points of tumor spheroid progression. Notably, our analysis allowed us to typify three time-invariant subgroups, each one with a particular functionality and genetic signature. As MCTS increases in size, oxygen and nutrients gradients are broadened, leading to a nutrient deprivation for cells closer to the spheroid core. Although we chose 19 days MCTS, they had nutrient deprivation even when they did not reach their maximum diameter. This phenomena leads us to believe that there were two ruling biological processes in our data, proliferation, and quiescence, one for each sampling time (Fig. 1b). In contrast with this hypothesis, we found three polarized states placed in independent subpopulations (Fig. 3a). Two of them majorly dominated by cells of a respective sample (clusters A and C) and a third one (Cluster B) with almost 30% of cells from both samples (Fig. 3b). Therefore, we concluded that there is not a unique biological process for a specific time point, several subpopulations coexist and might work together across time.

With the purpose to describe each subpopulation and their respective function, we performed gene-set enrichment and differentially expressed genes analysis. First, Cluster A enriched pathways comprised of several collections enlighten a strong activation of the immune system (Fig. 4). JAK/STAT signaling pathway has an anti-tumor defense and the maintenance of an active and long-term immune response^46^. As well, interferon response is praised as an important effector of anti-tumor immunity, capable of suppressing tumor growth^47^. However, interferon gamma (IFN-γ) facilitates tumor initiation and increases tumor fitness^47^. In addition, the immune response activation has been associated with metastasis and survival improvement driven by the induction of JAK/STAT3 pathway in several cancer types^48^. This immune activity is part of immune surveillance by presenting tumor-associated antigens, so tumor cells can be whipped out^49^. Nevertheless, immune cells recruitment is part of immune evasion^50^. As a first stage of it, tumor cells are recognized to be killed by T cells, NK cells, and macrophages. After the elimination stage, there is immunoediting in the major histocompatibility complex (MHC), followed by an evasion of tumor cells induced by immune selection^51–53^. In agreement with this, pathways related to antigen cross-presentation of the MHC1 were overrepresented in Cluster A (Supplementary Table S2). Although our model could only reproduce the beginning of the immune evasion process due to the lack of other immune response components, it gave useful information about their initiation. In addition, CXCL10 and S100 genes were linked to invasion and immune control (Fig. 7a). CXCL10 has a leading role in modulating cellular migration in the mouse breast cancer cell line 4T1^54^. Their immune role is mediated by the axis STAT1/IFN-γ leading to the polarization of macrophages to M1 and the recruitment of T and NK cells due to the secretion of proinflammatory cytokines^55,56^. On the other side, S100 genes codes for proteins of a calcium-binding receptor family. Evidence relates them to proliferation, metastasis, and immune evasion in breast cancer^57^. In ER+ breast cancer, S100A7 limits the proliferation and motility of tumor cells by downregulating the β-catenin/TCF4 pathway^58^. Moreover, the heterodimer S100A8/S100A9 enhances epithelial-mesenchymal transition. It binds with RAGE forming a stabilized SNAIL via NFκB, which provides a perfect niche for tumor cell invasion^59^. Additionally, S100A7, S100A8, and S100A9 recruit tumor-associated macrophages (TAM) to promote a microenvironment for immune evasion^60,61^. All together supports that Cluster A orchestrates immune evasion and invasion.

Results related to Cluster B are not clear enough to be associated with a unique function. Given the exploration we made, Cluster B shared characteristics from clusters A and C. Given their functionality closeness with the others clusters, a well-reasoned explanation is that this cluster is an intermediate state which leads to having a multipotent reservoir subpopulation. Moreover, this speculation should be validated in future work.

Regarding Cluster C, results indicated a classical cancer proliferating subpopulation. Cluster C overexpressed genes and pathways involved in the cell cycle progression, which is associated with the development and continued growth of cancers (Fig. 5b). Interestingly, it expressed genes with a double-edged function. ANLN, ANP32E, RACGAP1, and ARHGAP11A genes have an impact on the migration phenotype^40,41^ and they promote cellular proliferation. However, Cluster C was not associated with cell migration and invasion. This can be related to the fact that cells have latent phenotypes waiting for the correct stimuli to be manifested. This latency state of a particular population (in our experimental setup) may represent a transition pivot between the different clusters. On the whole, Cluster C is leading the important survival role of proliferating.

In terms of metabolism, the major functional differences were found between clusters A and C which impact the way they meet their energetic and metabolite demands. Contrastingly, results suggested Cluster A cells undergo glycolytic activity, while Cluster C cells overexpressed mitochondrial genes that conform respiratory complexes associated with OXPHOS (Supplementary Table S5). Although the glycolytic pathway is bio-energetically less efficient, it allows the adaptation to fluctuating oxygen availability and enables fast ATP production^43,62^, it is used in the presence or absence of oxygen and is associated with a malignant phenotype^63,64^. Moreover, some tumor cells use OXPHOS to produce energy and metabolites for pyrimidine biosynthesis^65^, which is related to the triphosphate metabolism, that maintains the correct levels of dNTPs for DNA replication and repair. dNTP synthesis is rate-limited by the reduction of ribonucleoside di- or tri- phosphates, carried out by RNR^66^. Cluster C overexpressed the RNR catalytic units (RRM1 and RRM2), and there is evidence that their inhibition is correlated to an increase in the migratory and metastatic potential^67,68^, as seen in Cluster A. We explain the representation of glycolytic pathways in our data due to the Warburg effect and to oxygen concentration on the spheroid layer. Given our results, we can expand the idea that glycolysis and OXPHOS are present in the same tumor accordingly with subpopulation functionality. So, these results agree that a common feature during cancer progression is metabolic rewiring^44,69^.

During cancer therapy, proper targeting of cell populations is one of the major challenges to optimize treatments. Overall, our study contributes with a proper heterogeneous model to identify targeted subpopulations. Development of cancer models capable of including heterogeneity is a major aim to balance between the optimal outcome of treatment and low risk of toxicity in patients. For example, although there are drugs that target IFN-γ/CXCL10 pathway^70^, there are other subpopulations of cells with different properties that lead to tumor survival; the inhibition of both OXPHOS and glycolysis simultaneously in MCF7 and MDA-MB-231 cells with tamoxifen and glycolysis inhibitors increases the induced cytotoxicity^71^. So, functional heterogeneity must be taken into account in future clinical therapies.

So far, we identified that Cluster A had invasive and immune-adaptive characteristics, Cluster B remained as a backup subpopulation, and Cluster C promoted proliferation. All three together coexist with synergism, a feature developed as a survival strategy^72^. And possibly all subpopulations are regulated by a master complex motif (Fig. 7b). At this stage, several questions remain open for future studies like the motif robustness, how all cells transmit information about their microenvironment and the way these key role players can be affected to have a more efficient therapy regime.

**Figure 8:**
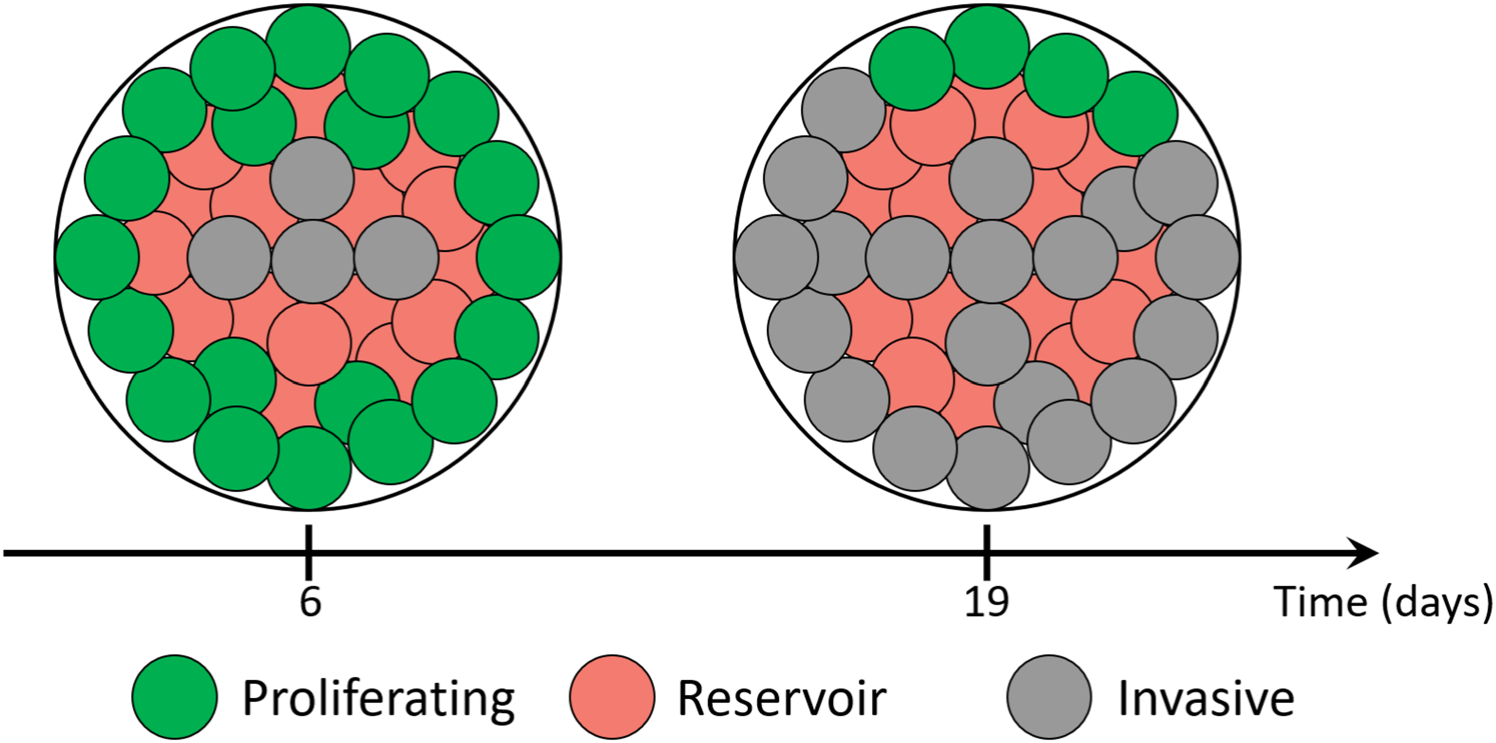
Change in the MCF7 MCTS subpopulations proportions over time. Every color represents a typified cluster.

Finally, one of our outstanding queries was the role of time in the MCF7 MCTS formation. We concluded that time determines the proportions of the three different subpopulations. Our results support the idea that metastasis is not the ultimate event in cancer, all processes have a systematic organization without a hierarchy as suggested in cancer development^72^. In agreement with our results, studies in glioblastoma solid tumors and cell lines demonstrate the existence of subpopulations with invasive and proliferative phenotype gene signatures^73,74^. Hence, the maintenance of clonal heterogeneity is an intrinsic property, where clones coordinate cooperatively growth, drug sensitivity, and motility^12^. Meanwhile, the proportion of proliferative and invasive subpopulations shifts with time, notably the reservoir remains constant (Fig. 8). The reservoir subpopulation might be an essential player in generating new tumors due to their shared characteristics with other clusters. Also, it is the most challenging to characterize because it does not have a differential marker even to develop a drug against him.

Single-cell studies had focused on describing intratumor heterogeneity by depicting the different cell subpopulations that structure a tumor. This global description had been conducted to find therapeutic targets and to make the first steps to unveil mechanisms in cancer development among cell subtypes. Here, we concluded that in an isogenic population a cooperative task stratification takes place. Each subpopulation is functionally non-redundant but complementary to support the tumor survival. Finally, it is important to highlight the main limitation of this study and their subsequent conclusions lay on their inability to include more realistic tumor micro environment variables; such as, vascularization, epithelial-mesenchymal transition, interaction with other cells in tissues and the constraints imposed by the tumor microenvironment. Moreover, the major difference given time progression is the proportion, so the occurrence probability of every function changes according to the tumoral microenvironment. The present study draws one of the heterogeneity foundations in cancer and opens several lines of research worth pursuing in future work. It would be worthwhile to consider the depicted functional heterogeneity in the development treatment schemes considering the existence of multiple populations with unique characteristics, with the ultimate goal to increase treatment efficacy.

## Methods

### Experimental procedures

#### Monolayer cell culture

Breast cancer cell line MCF7 (ATCC HTB-22TM) was growth in DMEM (ATCC 30-2002) containing 4 mM L-glutamine, 4500 mg/L glucose, 1 mM sodium pyruvate, and 1500 mg/L sodium. Media was supplemented with 10% v/v of FBS (ATCC 30-2020). Cells were incubated under a humidified atmosphere with 5% CO and 95% air at 37ºC. For all experiments, 70–80% confluent monolayer cultures with less than 9 passages were used. MCF7 cell line was validated using STR analysis.

#### Generation and disaggregation of Multicellular Tumor Spheroids (MCTS) Cultures from MCF7

The generation of spheroids was carried out using a liquid overlay technique. A single-cell suspension of MCF7 at a density of 1 × 10^6^ cells was loaded into 12.5 cm^2^ suspension culture flasks (UltraCruz sc-200257) containing 5 mL of L-15 media (ATCC 30-2008) supplemented with 5% v/v of FBS. Flasks were placed in an orbital incubator at 37ºC under constant orbital shaking of 59 rpm for 6 and 19 days^26^.

#### MCTS diameter distribution

The average and standard deviation of the MCTS Feret diameter were estimated by taking pictures directly to the spheroid flask during the culture kinetic. Pictures were obtained using a Nikon Eclipse TS 100 Inverted Microscope. We processed the images using MorphLibJ package^75^. The procedure above was performed for each biological replicate. We reported the average diameter, the standard deviation and the number of spheroids properly measured for each time point (Supplementary Table S1).

#### MCF7 MCTS disaggregation

For disaggregation, the 6 and 19 day-old spheroids were transferred to 1.5 mL tubes. The spheroids were washed with PBS 1X (VWR 97062-732). Accutase (Invitrogen 00-4555-56) was added and the reaction was carried out for 45 min at 37ºC with orbital shaking. Every 5 min the spheroids were gently pipetted. To ensure optimal disaggregation, Trypsin–EDTA (0.25% Trypsin, 1 mM EDTA) solution was added for no more than 5 min at 37ºC. The trypsin reaction was stopped by adding media with FBS in a 1:1 ratio. Cells were collected by centrifugation and suspended in 0.1% BSA in PBS (CST BSA #9998) solution.

#### MCTS culture time points selection

To capture cell heterogeneity four-time points were selected to explore the abundance of cells enriched on proliferative and quiescent populations. The first time point was 6 days of culture because MCTS were properly formed and aggregated. The next time point was selected exploiting MCF7 duplication time ~ 36 h, so the next point was 8. At 20 days of MCTS culture 90% of the sample had necrotic cores and maximum volume was reached, while samples taken from the 19th day still had necrotic cores, but in less proportion^26^. Therefore, we chose 24 and 48 h earlier (17 and 19 days) that the experimental model gets through its limits.

#### Sorting of cellular subpopulation from MCTS time points

After MCTS disaggregation of the 6, 8, 17, and 19 day-old spheroids. Cells were transferred to 1.5 mL tubes and suspended in 500 µL of PBS 1X (VWR 97,062–732). For MCTS fixation 500 µL of paraformaldehyde 4% w/v solution was added and the reaction was carried out for 10 min at room temperature. We removed the paraformaldehyde by washing 3 times with PBS 1X. MCTS samples were stored at 4 °C for a maximum period of 24 h after proceeding with permeabilization. For permeabilization, 450 µL of methanol 99.9% (MERK 34860-100ML-R) previously chilled in ice was added to the cells and the reaction was carried out for 30 min in ice. Before proceeding with immunostaining 1 × 10^6^ permeabilized cells were transferred to 15 mL centrifuge tubes (Corning CLS430055-100EA). Cells were washed twice with 3 mL of 5% w/v BSA-PBS solution. Cells from all-time point samples were immunostained by adding the proliferation (MKI67: Cell-signaling 11882) and quiescent marker (p27: Cell-signaling 12184) according to manufacturer procedure. Finally, cell sorting was done in a FACSAria Cell Sorter.

#### Isolation of single cells from MCF7 spheroids cultures

All scRNAseq procedures suggest a viable population higher than 90%. To this end, the viability of cells in suspensions was increased by removing dead cells with the dead cell removal kit (Miltenyi Biotec Inc. Order No. 130-100-008). Briefly, cells were centrifuged at 1500 rpm, resuspended and loaded into a LS column. Living cells were eluted with 12 mL of the binding buffer. Finally, cells were pelleted and resuspended in 1 mL of 0.1% BSA in PBS. Cell concentration and viability were assessed using trypan blue with the TC20TM automated cell counter.

#### Single-Cell RNA-Seq Library Construction and Sequencing

Cell suspension from the previous step was diluted to a final concentration of 2.5 × 10^6^ cells/mL with 0.1% BSA in PBS, and at least 6 µL of these final dilution suspension were needed as Input for the Bio-Rad ddSEQ Single-Cell Isolator (Illumina Bio-Rad SureCell WTA 3′ Library Prep). As viability and cell numbers are critical, three technical replicates of assessment were performed with the TC20 counter to ensure optimal conditions, i.e., cell viability > 90% and 2.5 × 10^6^ cell/mL. Then, two technical replicates, of 6 and 19 day-old spheroids respectively, were loaded onto the 4-sample cartridge for the ddSEQ. Isolation of single cells, library preparation, and sequencing were performed according to the manufacturer's reference guide (Illumina Bio-Rad SureCell WTA 3′ Library Prep Reference Guide). Sequencing was performed with a NextSeq 500/550 High Output Kit v2.5 (150 Cycles) to an attainable depth of 500,000 reads per cell.

#### Processing mRNA Sequencing Data

Raw sequencing data were processed and aligned to the human genome (hg19) following Romagnoli et al.^76^ and Tian et al.^77^.

### Data analysis

All statistical and bioinformatic data analysis was performed with R version 3.6. We excluded cells that had less than 1000 sequenced genes and genes with zero counts in all cells. Overall, we used 13,589 genes within 364 cells from the two-time points of spheroid progression. To estimate the number of cells required to represent the proportions shown in Fig. 1b, we used a sample-size statistical model for estimating the proportion of proliferative cells in spheroids. We estimated that a sample size of 115 and 255 cells was required to reproduce the proportions of proliferative cells observed at 6 and 19 days, respectively. We considered a margin of error of 5% with a confidence level of 90%. We experimentally obtained 134 and 230 cells for D6 and D19 spheroids, respectively. Thus, the number of cells experimentally measured has statistical support for representing the subpopulations.

#### Clustering and dimensionality reduction

Clustering was performed in three steps. First, we calculated the over-dispersion of each gene following Fan et al.^78^. Second, using the top N over-dispersed genes the distance matrix was computed as 1-Pearson-correlation of all cells. Finally, clustering was done with the following considerations.

Two matrices were used as input for cluster formation as parallel analyses. The first one was the distance matrix previously described; the second one was obtained by reducing the distance matrix to two dimensions with UMAP^29^ implemented in the “umap” R package with 300 iterations. To determine the clusters in both input matrices we used K-means and Expectation–Maximization (EM) algorithms. Optimum cluster number (k) for K-means was obtained using the maximum value of the second derivative of the sum of squared error for multiple k values on the elbow plot. EM algorithm was implemented with the “mclust” R package^79^.

#### Gene number selection

Each gene affects data clustering, as more considered it is harder to find similarities and differences between samples. However, considering a small amount of them wiped out subtle but significant properties in the sample. All the results were obtained by selecting the top 6,000 most over-dispersed genes. Therefore, selected genes comprised 68% of the total variance of data.

#### Pathway enrichment analysis

Pathway enrichment analysis was done using the Gene set enrichment analysis (GSEA)^30^ available at https://software.broadinstitute.org/gsea/. Considered gene sets were obtained from the MsigDB database (https://www.msigdb.org). We used the hallmarks^80^ and curated (C2) datasets. Statistical significance was assigned with an FDR < 0.05 and *p* value < 0.01. Since the analysis was done in pairwise comparisons, positive enriched pathways for one group were negative enriched pathways for the other and vice versa. Therefore, the negatively enriched pathways were implicitly considered.

#### Enriched map construction

Enriched maps were constructed with pathway enrichment analysis information. The description of every statistically significant enriched pathway was matched to find the most 3 representative words. Pathways with shared genes were favored to be considered in the same group. We computed the Jaccard index to find connections between different nodes, when it was greater than 0.4 a connection was settled. This threshold in the Jaccard index means that we set a relation when 40% of genes are contained in both gene sets. This value is recommended in Cytoscape software. A preliminary map was constructed with Cytoscape and refined with our own algorithms.

#### Differential expression analysis

Differential expression analysis was performed with package SCDE^81^ for R, using a |log2(FC)|> 1.5 and a *p* value < 0.01. Heatmaps were constructed taking the logarithmic transformation of the genes count table plus one.

#### Correlating enriched pathways with differentially expressed genes

The link between analyses was done in two steps: First, we extracted the most recurrent leading edges genes of the gene set analysis for every collection described in the enriched maps. Second, we quantified the number of hits for the differentially expressed genes into the most representative genes for each collection of step one, genes with the most hits were set as key genes.

#### Regulation Motif

We used IPA software which correlates desired genes into a built-in scientific literature database. We ranked the expression data of every comparison by log2(FC) and *p* value, and subsequently, we loaded the data into IPA core analysis. Taking hand of the IPA upstream regulators tool, we associated upstream molecules to the expression data. Then, we took only the regulators that were interconnected with several genes.

## Supplementary information

Supplementary Figures.

Supplementary Table 1.

Supplementary Table 2.

Supplementary Table 3.

Supplementary Table 4.

Supplementary Table 5.

Supplementary Table 6.

Supplementary Table 7.

Supplementary Table 8.

Supplementary Table 9.

## Data Availability

Raw data are available through the Gene Expression Omnibus (GEO) with accession number GSE145633. All code used for analysis is available at https://github.com/resendislab/scPipeline.
