# Supplementary material for: Unveiling functional heterogeneity in breast cancer multicellular tumor spheroids through single-cell RNA-seq
Source: Sci Rep. 2020 Jul 29;10:12728. doi: 10.1038/s41598-020-69026-7 (PMC7391783; doi:10.1038/s41598-020-69026-7)
Supplement: Supplementary file 1 — Supplementary Figures. [file 41598_2020_69026_MOESM1_ESM.pdf]

Supplementary Material

Unveiling Functional Heterogeneity in Breast Cancer Multicellular Tumor Spheroids through Single-Cell RNA-seq.

Erick Andrés Muciño-Olmos*, Aarón Vázquez-Jiménez*, Ugo Avila-Ponce de León, Meztli Matadamas-Guzman, Vilma Maldonado, Tayde López-Santaella, Abrahan Hernández-Hernández** & Osbaldo Resendis-Antonio **

*Co-first authors of this manuscript.

** Corresponding authors: oresendis@inmegen.gob.mx, abrahan.hernandez@himfg.edu.mx

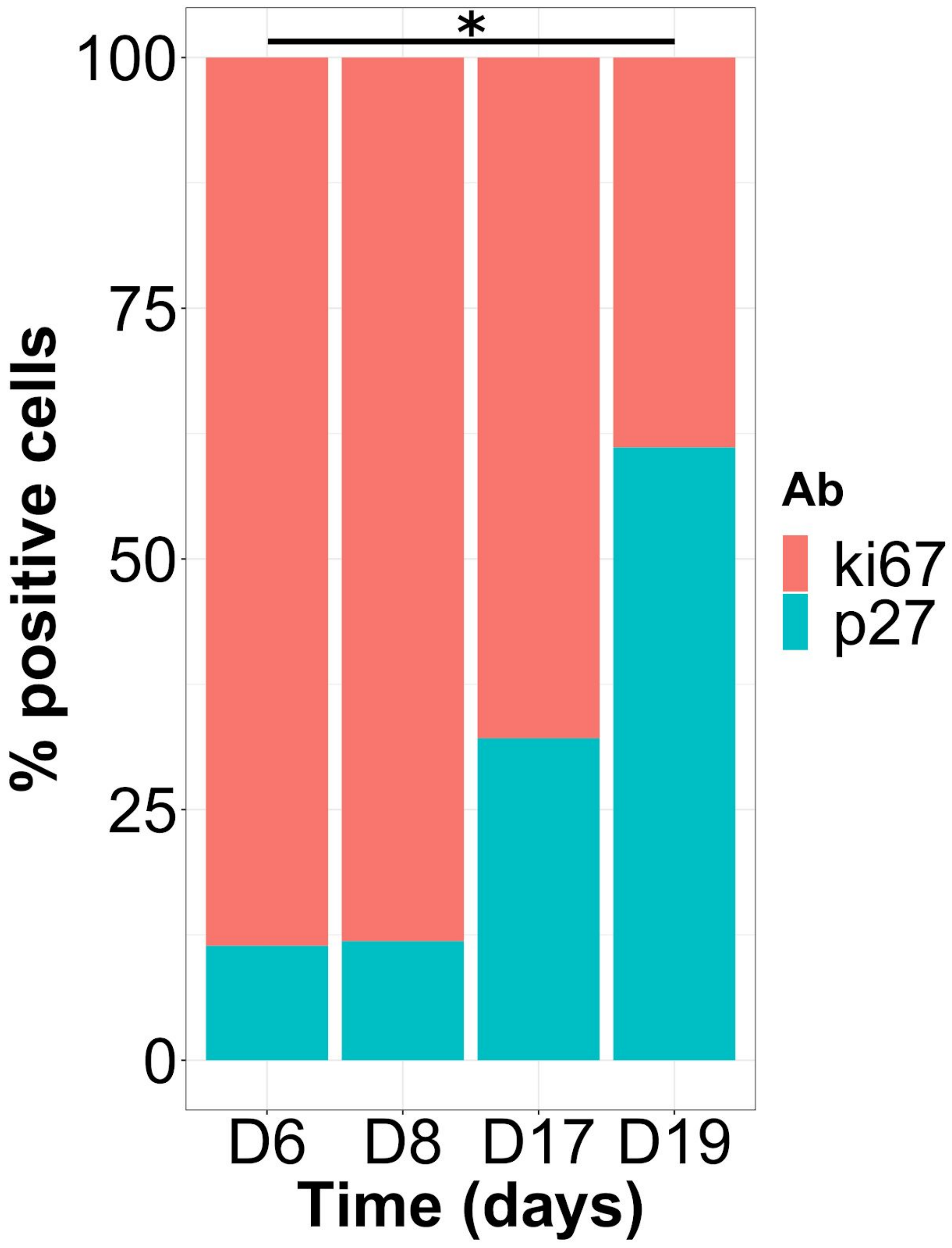

Figure S1: Immunophenotyping with flow cytometry analysis with KI67 and p27 markers given four temporal conditions, days 6, 8, 17 and 19. Expression of both markers for the D6vsD19 comparison shows statistical difference with a p-value<0.05 using a non-paired t-student test with equal variances. P-values for p27 and ki67 markers are 0.036 and 0.004, respectively. A Levene's Test was performed to test the equality of variance, non-significative results were found.

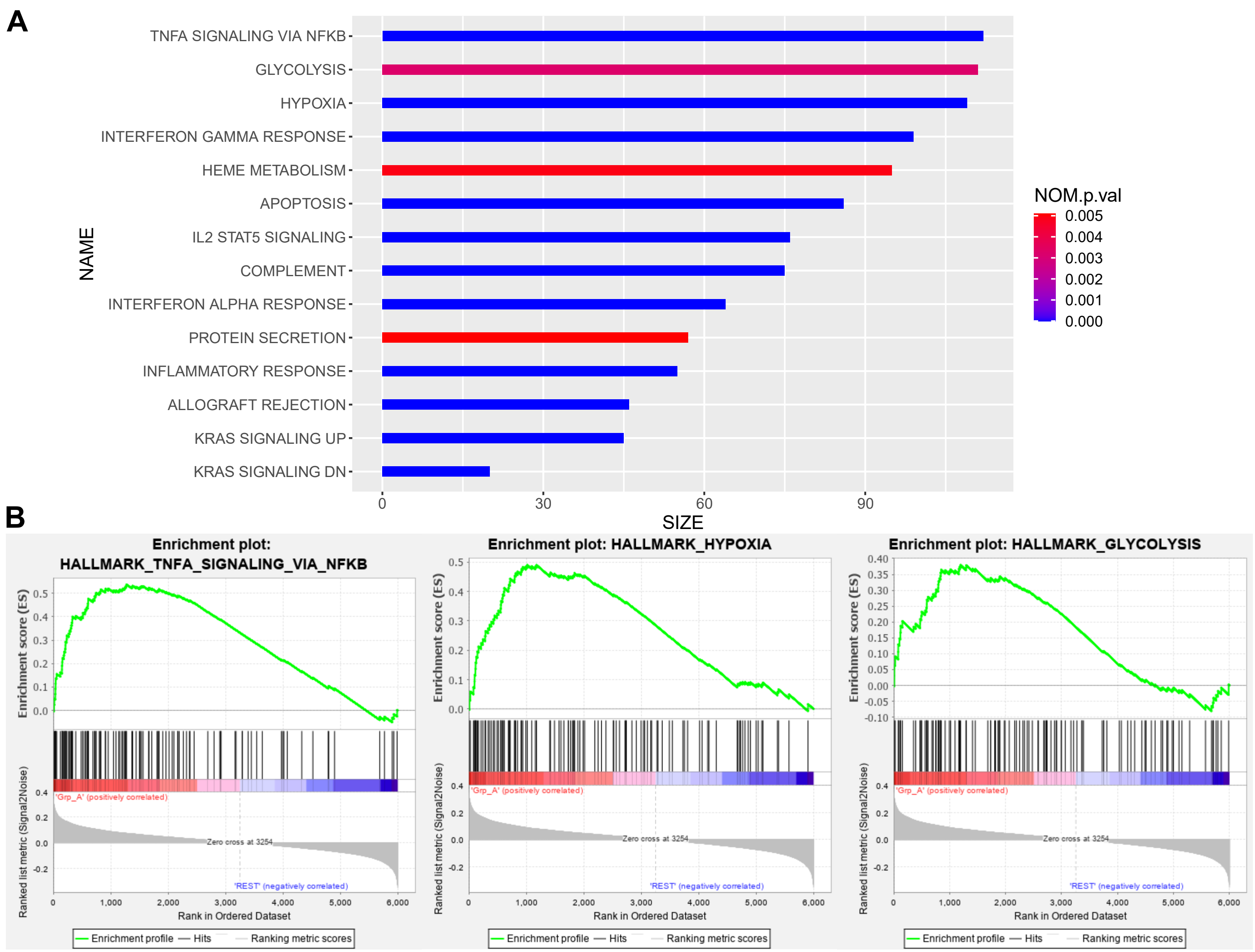

Figure S2: A. Cluster A barplot for the enriched gene sets of the MSigDB hallmark collection[1] with an FDR<0.05 and a p-value<0.01. B. Enriched plots for the top three enriched gene sets.

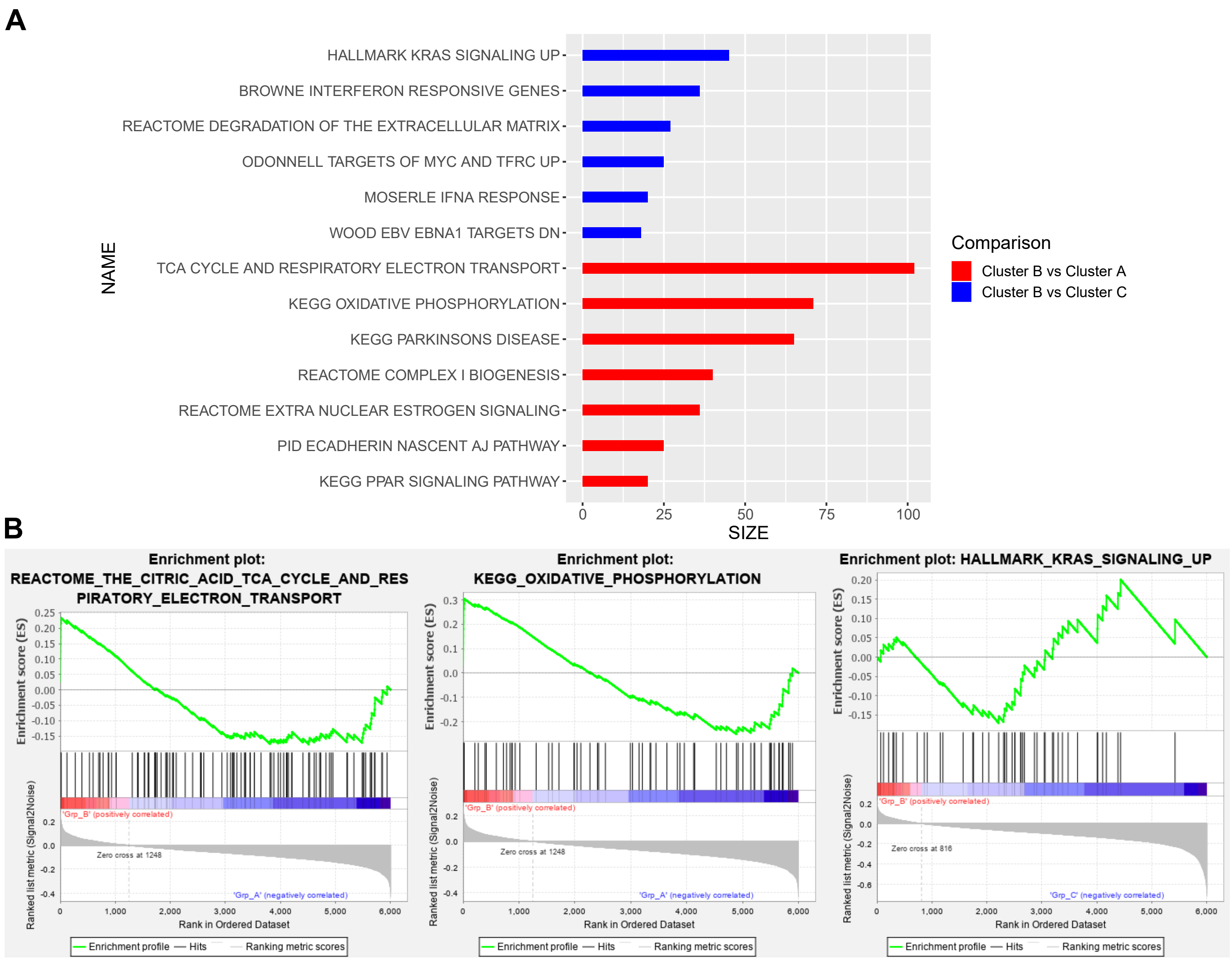

Figure S3: A. Cluster B barplot for the enriched gene sets for both comparisons with an FDR<0.05 and a p-value=0. B. Enriched plots for the top three enriched gene sets of both comparisons.

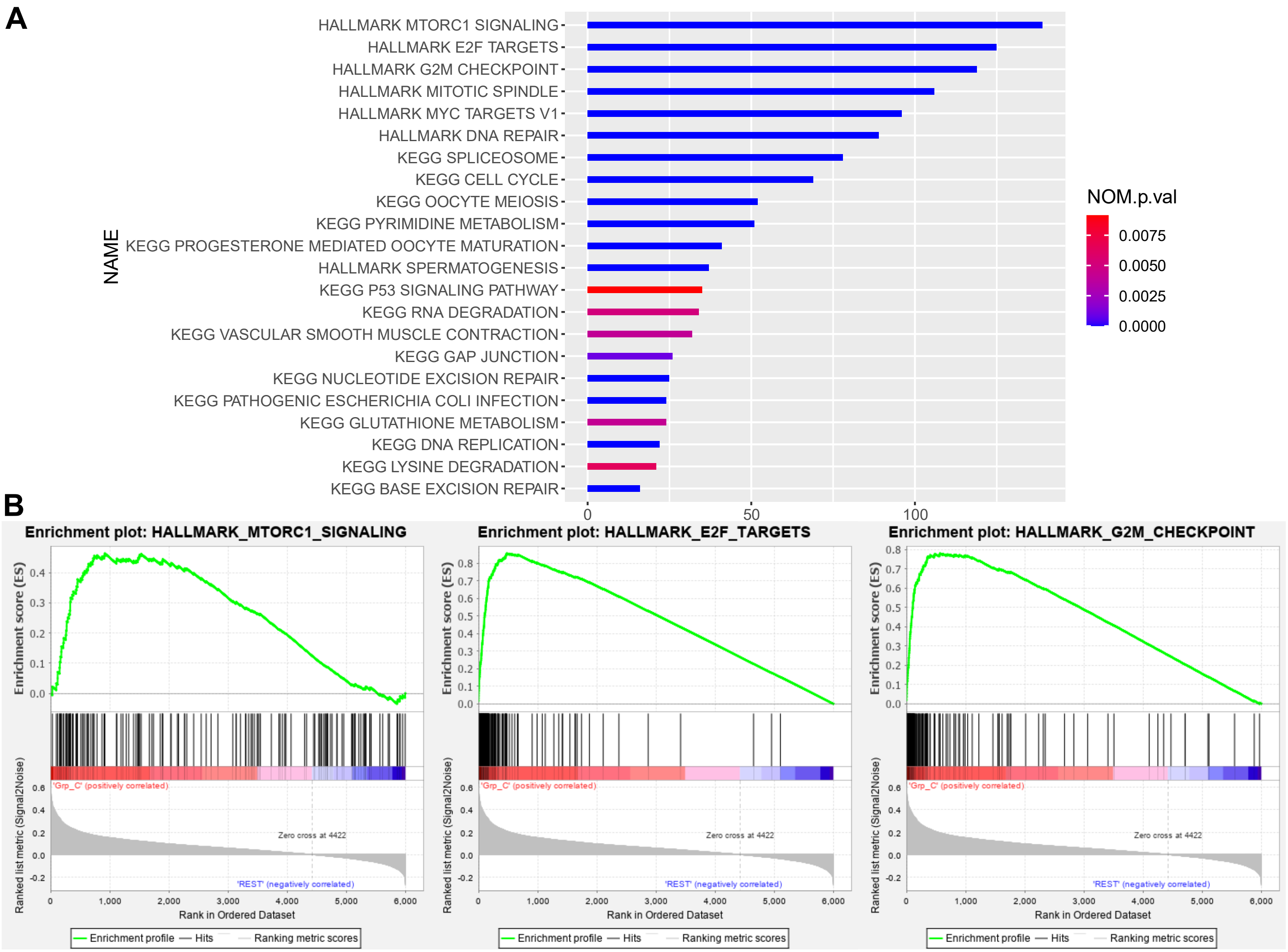

Figure S4: A. Cluster C barplot for the enriched gene sets of the MSigDB hallmark[1] and KEGG[2] collections with an FDR<0.05 and a p-value<0.01. B. Enriched plots for the top three enriched gene sets.

References

1. Liberzon, A. *et al.* The Molecular Signatures Database Hallmark Gene Set Collection. *Cell Systems* **1**, 417–425 (2015).
2. Minoru, K. & Susumu, G. KEGG: Kyoto Encyclopedia of Genes and Genomes. *Nucleic Acids Research* **28**, 27–30 (2000).
